# Kir6.1, a component of an ATP-sensitive potassium channel, regulates natural killer cell development

**DOI:** 10.3389/fimmu.2024.1490250

**Published:** 2024-12-02

**Authors:** Natalie Samper, Lilja Hardardottir, Delphine M. Depierreux, Soomin C. Song, Ayano Nakazawa, Ivan Gando, Tomoe Y. Nakamura, Andrew M. Sharkey, Carla R. Nowosad, Stefan Feske, Francesco Colucci, William A. Coetzee

**Affiliations:** ^1^ Department of Pathology, NYU Grossman School of Medicine, New York, NY, United States; ^2^ Department of Obstetrics and Gynecology, University of Cambridge, Cambridge, United Kingdom; ^3^ Department of Pharmacology, Wakayama Medical University, Wakayama, Japan; ^4^ Department of Pathology, University of Cambridge, Cambridge, United Kingdom; ^5^ Department of Neuroscience & Physiology, NYU Grossman School of Medicine, New York, NY, United States; ^6^ Department of Biochemistry and Molecular Pharmacology, NYU Grossman School of Medicine, New York, NY, United States

**Keywords:** innate immunity, NK cells, ion channels, potassium channels, ATP-sensitive potassium channels, NK cell development

## Abstract

**Introduction:**

Involved in immunity and reproduction, natural killer (NK) cells offer opportunities to develop new immunotherapies to treat infections and cancer or to alleviate pregnancy complications. Most current strategies use cytokines or antibodies to enhance NK-cell function, but none use ion channel modulators, which are widely used in clinical practice to treat hypertension, diabetes, epilepsy, and other conditions. Little is known about ion channels in NK cells.

**Results:**

We show that *Kcnj8*, which codes for the Kir6.1 subunit of a certain type of ATP-sensitive potassium (K_ATP_) channel, is highly expressed in murine splenic and uterine NK cells compared to other K^+^ channels previously identified in NK cells. *Kcnj8* expression is highest in the most mature subset of splenic NK cells (CD27^-^/CD11b^+^) and in NKG2A^+^ or Ly49C/I^+^ educated uterine NK cells. Using patch clamping, we show that a subset of NK cells expresses a current sensitive to the Kir6.1 blocker PNU-37883A. *Kcnj8* does not participate in NK cell degranulation in response to tumor cells in vitro or rejection of tumor cells *in vivo*, or IFN-γ release. Transcriptomics show that genes previously implicated in NK cell development are amongst those differentially expressed in CD27^-^/CD11b^+^ NK cells deficient for *Kcnj8*. Indeed, we found that mice with NK-cell specific *Kcnj8* gene ablation have fewer CD27^-^/CD11b^+^ and KLRG-1^+^ NK cells in the bone barrow and spleen.

**Discussion:**

These results show that the K_ATP_ subunit Kir6.1 has a key role in NK-cell development.

## Introduction

Natural Killer (NK) cells have a key role in immune surveillance, anti-tumor immunity and anti-viral immunity by identifying and eliminating transformed or infected cells. NK cells lyse these target cells through regulated exocytosis of specialized secretory lysosomes, termed cytotoxic granules, which contain proteins such as perforin, granzymes, and the Fas ligand. NK cells also release chemokines and cytokines, including interferon-γ (IFN-γ) that is critical for cancer immune surveillance (1) and provides feedback to other immune cell types, thus modulating immune responses. Thus, cytotoxicity and secretion of cytokines and chemokines are two independent effector functions that largely account for their antiviral and antitumor activities. A special type of NK cell in the uterus (uNK) mediates physiological vascular changes that enable the placenta to sustain healthy fetal growth (2). Understanding the mechanisms underlying NK cell development, education and activation is essential for developing therapeutic strategies targeting NK cells.

In C57BL/6 mice, the NK cell maturation program in bone marrow is marked by sequential surface expression of integrin alpha M (*Itgam*), also known as CD11b, MAC-1 or CR3, and the TNF-superfamily CD27 receptor (3). CD27^−^/CD11b^−^ double negative (DN) cells develop in the bone marrow and are the most immature. After the acquisition of CD27 and CD11b, expression of S1P5R enables the CD27^+^/CD11b^+^ double-positive (DP) NK cells to migrate from the bone marrow to the periphery, including the blood, spleen, and other organs. With further maturation, CD27 expression is lost and CD27^-^/CD11b^+^ remains as the most mature population with the highest cytotoxic potential.

Later in development, NK cell acquire inhibitory receptors that recognize self-MHC class I molecules. These receptors include killer cell immunoglobulin-like receptors (KIRs) in humans, Ly49 receptors in mice, and CD94/NKG2A in both species (4, 5). Together with signals elicited by activating NK cell receptors, they regulate NK cell activation. NK-cell education occurs through homeostatic interactions between self MHC class I molecules and NK cell inhibitory receptors (6), ensuring that NK cells effectively distinguish between healthy cells and those with inadequate MHC class I expression due to infection or transformation. When NK cells interact with cells expressing self-MHC class I molecules, inhibitory signals are transmitted through these receptors, preventing NK cell cytotoxicity against healthy cells. NK cells can directly recognize cells with aberrant expression of MHC class I molecules (“missing self”) or cells expressing stress-induced ligands or viral proteins (“induced self”) that directly engage the activating receptor NKG2D. Engagement of activating receptors in the absence of ligands for inhibitory receptors initiates signaling pathways leading to NK cell activation. NK cell activation can also be triggered by cytokines such as interleukin-2 (IL-2), IL-12, IL-15, IL-18, and interferon-alpha (IFN-α), the latter induced experimentally in mice by administration of the viral RNA mimic polyinosinic:polycytidylic acid (poly I:C).

Ion translocating proteins regulate immune cell function. There are hundreds such proteins that control the movement of ions such as Na^+^, K^+^, Cl^-^, Ca^2+^, Mg^2+^, Zn^2+^ across the plasma membrane. Although extensively studied in the nervous, endocrine, and cardiovascular systems, the roles of this important class of proteins are scantly studied in immune cells (7). In T cells, B cells, monocytes, macrophages, dendritic cells and neutrophils, roles have been described for certain K^+^ channels (KCa3.1 and Kv1.3), Ca^2+^ channels (mainly ORAI1/STIM1), P2X receptors, and some members of the transient receptor potential (TRP) family (8). The involvement of some of these channels in immunity is highlighted by inherited gene variants (channelopathies) in immunodeficiency disorders. By contrast, ion channels, exchangers and pumps in NK cells are poorly characterized. There are only a few studies describing roles of ion channels in NK cells. The K^+^ concentration and some ion channel blockers can regulate human NK cell-mediated killing (9–11). K^+^ channels in NK cells have been described, including voltage-gated K^+^ channels (12, 13), Ca^2+^-activated K^+^ channels (14, 15), two-pore domain K^+^ channels (16), Ca^2+^-permeable cation channels (17), intracellular Ca^2+^-permeable channels (18, 19), and store-operated Ca^2+^ entry (SOCE) channels (20). The lack of a complete understanding of the role of ion translocating mechanisms in NK cells is a major gap in our knowledge of immunity. This is also a possible missed opportunity for the development of novel therapeutic approaches in inflammatory and anti-cancer therapy.

ATP-sensitive K^+^ (K_ATP_) channels are regulated by intracellular nucleotides (ATP and ADP). As such, they couple the intracellular energy metabolic state to membrane excitability and secretory events (21, 22). In the pancreatic β-cell, for example, K_ATP_ channel activity initiates the release of insulin from intracellular granules after a meal (23), whereas in the heart they participate in stress responses (21). The K_ATP_ channel is a tetrameric structure composed of four pore-forming subunits (Kir6.1 or Kir6.2) in association with four sulphonylurea receptors (SUR1 or SUR2) (24, 25) (**Supplementary Figure S1**). *KCNJ8* and *ABCC9* are genes adjacent to each other on human chromosome 12 and respectively code for Kir6.1 and SUR2 subunits, whereas the adjacent *KCNJ11* and *ABCC8* on chromosome 11 respectively code for Kir6.2 and SUR1 subunits. The aim of this study was to characterize the expression of *Kcnj8* in mouse NK cells and to determine whether *Kcnj8* has a role in NK cell development and function. We found *Kcnj8* to be expressed at high levels in a mature subset of mouse NK cells. NK-cell specific *Kcnj8* gene ablation did not interfere with the basic NK-cell functions that we studied, but impeded full NK cell maturation.

## Methods

### Generation of NK cell-specific Kir6.1 knockout mice

The mouse model was generated by inGenious Targeting Labs Inc. (iTL; Stony Brook, New York). In brief, a 10.4 kb region used to construct the targeting vector was first subcloned from a positively identified C57BL/B6 BAC clone (RPCI-23: 108N15) BAC clone. The region was designed such that the short homology arm (SA) extended ~1.76 kb 3’ to exon 2. The ~7.2 kb long homology arm (LA) ends 5’ to exon 2. The loxP/FRT flanked Neo cassette was inserted on the 3’ side of exon 2 and the single loxP site is inserted on the 5’ of exon 2. The target region was 1,460 bps and includes exon 2. The targeting vector was linearized with NotI and transfected by electroporation of BA1 (C57BL/6 x 129/SvEv) hybrid embryonic stem cells. After selection with G418 antibiotic, surviving clones were expanded for PCR analysis to identify recombinant ES clones. Hybrid positive ES cells were injected into C57BL/6 blastocysts. The Neo cassette was removed by breeding to FLP deleter mice (Jackson stock #005703), followed by backcrossing to a C57BL/6 background for >10 generations to achieve congenicity. To target NK cells, we used inducible NKp46-CreERT2 mice from Dr. Lewis Lanier (UC San Francisco, USA) (26) that were intercrossed with Rosa26-tdTomato C57BL/6 mice. The Kir6.1^flx/flx^ mice were crossed with NKp46-CreERT2 and bred to homozygosity. Kir6.1 was deleted in NK cells by treating mice with tamoxifen (IP injection of 2 mg tamoxifen in corn oil, daily for 4 days). In some experiments we used a similar mouse model in which exon 2 of *Kcnj8* is flanked by loxP sites (provided by Dr. Andrew Tinker) that was crossed with constitutively expressed NKp46-Cre mice provided by Dr. Eric Vivier (Aix-Marseille University, France) (27).

### Isolation of mouse splenic NK cells

All animal handling procedures were in accordance with National Institutes of Health guidelines and were approved by the Institutional Animal Care and Use Committees of New York University School of Medicine and the University of Cambridge. Male and female C57BL/6 mice aged 8-12 weeks were housed in pathogen-free conditions, before being euthanized and collection of organs such as the spleen, brain or bone marrow. For bone marrow cells, the femur was collected, cells were flushed with PBS in a syringe and passed through a 70 µm cell strainer followed by washing with PBS. Cells from peritoneal cavity were aspirated with a syringe and washed with PBS. In some experiments, spleens were harvested in media (RPMI + 10% FBS) on ice and passed through 70 µm cell strainer along with media and centrifuged. Erythrocytes were lysed with 1xRBC lysis buffer (Ebioscience) for 3 min at RT. Splenocytes were washed 2x with media and passed again through 70 µm cell strainer. In other experiments, spleens were dissociated with the gentleMACS spleen disassociation kit (Miltenyi Biotec). Cells were then filtered, erythrocytes were depleted via isotonic hemolysis, and NK cells were isolated using a negative selection method (NK Cell Isolation Kit, Miltenyi Biotec) following the manufacture’s guidelines. In this negative selection method, non-NK cells, i.e. T cells, dendritic cells, B cells, granulocytes, macrophages, and erythroid cells are magnetically labeled with a cocktail of biotin-conjugated antibodies and anti-biotin MicroBeads, and then removed from isolated splenic cells using magnetic columns.

### Single cell RNA seq of splenic NK cells

Splenic NK cells from 3 wildtype mice were isolated via negative selection as previously described. Cells were lysed and RNA was obtained via PicoPure™ RNA Isolation Kit (Applied Biosystems). Lipid-based cell multiplexing was used in order to combine NK cell populations isolated from 3 mice. Single cell RNA sequencing (RNA seq) was coordinated by NYU Langone’s Genome Technology Center (RRID: SCR_017929). We used the Chromium Next GEM Single Cell 3' Reagent Kit v3.1 (10x Genomics) to perform gel beads-in-emulsion (GEM) generation and barcoding, Post GEM-reverse transcriptase (RT) cleanup and cDNA amplification, and 3’ gene expression library construction. Sequencing was performed by NYU Langone’s Genome Technology Center using an Illumina NovaSeq 6000 instrument. Demultiplexing raw base call (BCL) files into FASTQ format was performed using Cell Ranger software (10X Genomics). The latter software was also used for alignment, filtering, barcode counting, and UMI counting to generate feature-barcode matrices. The estimated number of cells was 16,384, with 72,726 mean reads per cell (2,047 median reads per cell). Of a total of 1,191,540,284 reads, 96.6% was mapped to the mm10 mouse genome.

Further analysis including quality filtering, the identification of highly variable genes, dimensionality reduction, standard unsupervised clustering algorithms, and the discovery of differentially expressed genes was performed using the Seurat R package (28). We filtered out cells with unique feature counts (genes) over 5,000 or less than 200. We also filtered out cells with more than 15% mitochondrial gene counts. Global-scaling normalization (“LogNormalize”) was performed that normalizes the feature expression measurements for each cell by the total expression, multiplies this by a scale factor (10,000) and log-transforms the result. Linear transformation (scaling) was performed, followed by dimensional reduction using a principal component analysis (PCA). Nearest-neighbor analysis, constrained to a maximum of 20 dimensions, was performed, followed by a graph-based approach to cluster cells at a resolution value of 0.5. Clusters were displayed after non-linear dimensional reduction with t-distributed stochastic neighbor embedding (tSNE) and uniform manifold approximation and projection (UMAP) methods. Differentially overexpressed features (cluster biomarkers) were determined using the ‘FindAllMarkers’ function at a minimum fold difference (log-scale) of 0.25.

### Bulk RNA seq of mouse splenic NK cells

Splenocytes were isolated from wild-type mice, and Kir6.1^flx/flx^ x NKp46-CreERT2 mice immediately following 4 days of tamoxifen induction (n=2 each). Cells were first gated for NK cell markers NK1.1 and NKp46, then sorted into four pools (CD27^-^ /CD11b^-^, CD27^+^ /CD11b^-^, CD27^+^ /CD11b^+^, and CD27^-^ /CD11b^+^). RNA was isolated with the RNeasy Mini Kit (Qiagen). RNA extractions were quantified using RNA Pico Chips (Agilent, Cat. 5067-1513) on an Agilent 2100 BioAnalyzer. cDNA was synthesized using the Takara SMART-Seq HT kit (Cat. 634438) with 0.5 ng of RNA input and 14 amplification cycles. The Nextera XT DNA library Kit (Illumina, Cat. FC-131-1096) was used to construct sequencing libraries with 0.25 ng cDNA input and 11 cycles amplification. Final libraries were visualized using High Sensitivity DNA ScreenTape (Agilent, Cat. #5067-5584) on the Agilent Tapestation 2200 instrument. Quant-It (Invitrogen, Cat. P11495) was used for final concentration determination and libraries were pooled equimolar. Paired-end 50 cycle RNA sequencing was performed by NYU Langone’s Genome Technology Center (RRID: SCR_017929) using a single lane of a 10B 100 Cycle Flowcell using an Illumina NovaSeq X-Plus instrument. Data analysis was performed on BigPurple, a high-performance computing cluster available through NYU Langone’s High Performance Computing (HPC) Core. Quality control of the paired RNA fastq files was performed with fastqc. No end-trimming was needed. Reads were aligned to the mm10 mouse genome using STAR and index files were produced with Samtools. The GeneCounts option of STAR was used to count number reads per gene while mapping. Off-line analysis was performed with R. Individual “ReadsPerGene.out.tab” files were combined. Gene expression analysis was performed with the DESeq2 package (version 1.44.0), which included filtering low expressing genes, calculating normalized gene counts, calculating the between-sample distance matrix, principal component analysis, and performing a differential expression analysis. Gene ontology analysis was performed with Enrichr (29). The scripts used for analysis are available upon request.

### Bulk RNA seq of uNK cells

At gestation day 9.5-10.5, C57BL6 mice were culled by cervical dislocation to collect the pregnant uterus which was enzymatically and mechanically processed into a single suspension as previously described (30). Single cell suspensions were labeled with viability dye Zombie Aqua Fixable Viability Kit according to the manufacturer’s protocol. Fc receptors were blocked with anti-CD16/32 mAb (Trustain Fcx) for 10 min at 4°C. Cell surface antigens were labeled for 30 min at 4°C in the dark, with the indicated cocktail of antibodies (**Supplementary Table S1**). Cells were subsequently washed twice in PBS, and collected by centrifugation at 400 g for 5 min at 4°C. Live single cells, negative for CD3, CD4, CD8, CD19, CD11b high, and positive for CD45 and Nkp46 were defined as NK cells. NK cells were further gated according to their relative expression of LY49C/I and NKG2A. Three cell populations were collected (Ly49C/I neg, NKG2A neg; LY49C/I neg, NKG2A pos; LY49C/I pos NKG2A neg). Double positive cells were not collected due to limited sample size. Samples were stored at -80°C until RNA extraction. RNA Extraction and Processing library construction was performed as previously described (31). Reads were trimmed using TrimGalore v0.5.0. and were mapped using STAR v2.7.1. to the Ensembl Mus_musculus GRCm38 (release 103) reference genome. Differential gene expression analysis was done using the counted reads and the R package edgeR version 3.26.5 (R version 3.6.1). The threshold was set to include genes with at least 5 counts per million reads mapped and detected in at least half the samples to allow for on/off expression pattern detection. Correction for GC content and gene length bias was applied using CQN bioconductor package (version 1.30.0). The resulting p-value was corrected for multiple hypothesis testing using the Benjamini-Hochberg method.

### qRT-PCR experiments

Splenic NK cells isolated with a negative selection method were used. First strand cDNA synthesis was performed with SuperScript™ IV (ThermoFisher Scientific) using random primers. Primers used for PCR spanned the boundary of exons 1 and 2 of *Kcnj8* (1585F: 5’-CACAAGAACATCCGAGAGC-3’ and 1585R: 5’-GGGCATTCCTCAGTCATCAT-3’), which produced a 320bp amplicon. The cycling conditions consisted of heating to 95°C for 3 min, 28 cycles consisting of 94°C for 30s, 55°C for 45s, 72°C for 1 min; followed by 1 min extension at 72°C. Amplicons were resolved by 1% agarose gel electrophoresis and visualized with ethidium bromide.

### Flow cytometry

Single cell suspensions were obtained from organs. Briefly, cells were blocked with TruStain FcX™ (anti-mouse CD16/32) antibody (Biolegend) according to the manufacturer’s protocol. Cells were stained with relevant antibodies for 30 min on ice in the dark followed by two washes with PBS. When detecting intracellular antigens, the cells were fixed and permeabilized with eBioscience Fixation/Permeabilization kit, according to manufacturer’s protocol prior to staining with intracellular antibodies. Data was acquired on a Cytek Aurora instrument.

Antibodies used for flow cytometry: Fixable Viability Dye eFluor™ 780 (eBioscience), CD3 BV785 (Biolegend), NK1.1 PE (Biolegend), CD11b BV421 (BD Biosciences), CD27 BV605 (BD Biosciences), CD49b Pe-Cy7 (Biolegend), DNAM1 AF647 (Biolegend), KLRG1 FITC (eBioscience), h2-kb BV421 (Biolegend), CD107a FITC (BD Biosciences).

In some experiments, either whole spleen or isolated NK cells were incubated in PEB buffer (PBS+0.5% BSA+2mM EDTA) with TruStain FcX™ (anti-mouse CD16/32) antibody (Biolegend) on ice for 5 minutes. Cells were then stained with the appropriate conjugated surface markers in PEB buffer protected from light at 4°C for 20 min. In the case of intracellular staining, cells were incubated with IC Fixation Buffer (eBioscience) for 30 min and then Permeabilization Buffer (eBioscience) with addition of conjugated antibody overnight. Anti-mouse antibodies used cytometry: NKp46 (29A1.4) BV421 (Biolegend), NK1.1 (PK136) APC-Cy7 (Biolegend), NK1.1 (PK136) PE (Biolegend), CD11b (M1/70) APC (Biolegend), CD11b (M1/70) AF488 (Biolegend), CD27 (LG.3A10) APC-Cy7 (Biolegend), CD27 (LG.3A10) AF647, and CD107a (1D4B) APC/Fire750 (Biolegend).

### Degranulation assay with target cells

In some experiments, NK-specific *Kcnj8* KO and WT mice were injected intravenously with 100 µg poly I:C (Miltenyi Biotec) in 200 µL sterile PBS. After 24 h, splenocytes were harvested and NK cells were enriched with a negative NK cell isolation MACS kit, according to the manufacturer’s protocol. NK cells were co-incubated with RMA, RMA-S or RMA-RAE1y target cell lines in 1:1 ratio and treated with eBioscience Protein Transport Inhibitor Cocktail, according to manufacturer’s protocol. Cells were incubated for 2h at 37°C, and the frequency of CD107a expression in NK cells was determined with flow cytometry. In other experiments, after 4 days of tamoxifen injection, NK-specific *Kcnj8* KO and WT mice were injected intraperitoneally with 100 µg poly I:C (Miltenyi Biotec) in 200 µL sterile PBS. After 18h, splenocytes were harvested and NK cells were enriched with a negative NK cell isolation MACS kit, according to the manufacturer’s protocol. NK cells were co-incubated with YAC-1 target cells in 1:1 ratio with a test of conjugated CD107a antibody and treated with BD Biosciences GolgiStop Protein Transport Inhibitor Cocktail containing monensin, according to manufacturer’s protocol.

### IFN-γ secretion

Splenic NK cells were isolated by negative selection from WT and KO mice, and cultured in RPMI-1640 with 10% fetal bovine serum, 1% penicillin, 1% streptomycin, and 50 mM 2-mercaptoethanol. Cells were treated with IL-15 (10 ng/ml) for 18 h and two other groups were stimulated with IL-15 (10 ng/ml) plus IL-18 (100 ng/ml) for 18 h, or with PMA (80 nM) and ionomycin (1.3 µM) for 18 h. The INF-γ content in the media was measured by ELISA (Mouse IFN-gamma DuoSet ELISA; R&D Systems).

### *In vivo* killing assay with target cells

Three cell lines, RMA, RMA-S and RMA-RAE1y, were cultured *in vitro* in RPMI plus 10% FBS at 37°C. RMA cells were stained with Far Red Cell Proliferation kit (Thermo Fischer) while RMA-S and RMA-RAE1y cells were stained with CellTrace CFSE Cell Proliferation kit (ThermoFisher), according to manufacturer’s protocol. Equal amounts of each cell line were harvested and combined. The cells were administered intraperitoneally (in sterile PBS) to NK-specific *Kcnj8* KO mice and WT mice. The cells were harvested from the peritoneal cavity 48 h later, washed and quantified with flow cytometry by assessing CFSE, FarRed and h2-kb (to separate RMA-S and RMA-RAE1y).

### RNAscope *in situ* hybridization

Mouse spleens were carefully handled and fixed for 36-48h at room temperature with 4% paraformaldehyde (at pH 7.4 prepared in PBS) in a sufficiently large volume (at least 10x spleen volume) with gentle rocking ensuring that no air bubbles were in contact with the spleen. Tissue was processed by NYUMC’s Experimental Pathology Research Laboratory (RRID: SCR_017928), which included paraffin embedding, sectioning (3 µm thickness) and the fluorescence RNA *in situ* hybridization assays with RNAscope^®^ technology using the RNAscope Multiplex Fluorescent V2 Assay (Advanced Cell Diagnostics, Newark, CA). Multispectral imaging was performed with a Akoya/PerkinElmer Vectra^®^ instrument. The RNAscope™ LS 2.5 probes (Advanced Cell Diagnostics, Newark, CA) used included Mm-Kcnj8 (#411398), Mm-Abcc9 (#411378), Mm-Ncr1-C2 (#501729), Mm-Cd3e-C3 (#314728), and Mm-Adgre1-C4 (#460658). Opal dyes (Akoya Biosciences, MA) were used for secondary staining as follows: Opal 690 for C1 and Opal 570 for C3. DAPI was used for nuclear staining.

The spectrally unmixed fluorophores were demultiplexed using InForm software (Akoya/PerkinElmer). Cell segmentation, based on DAPI nuclear staining, and mRNA dots/cell were analyzed using HALO version 3.6 (Indica Labs, Albuquerque, NM). For nuclear detection the Nuclear Segmentation AI classifier was further trained with nuclei from the images used to quantify and labeled as ‘Nuclear_Segmentation_Mouse_spleen’. Each cell was defined as the nuclear segmentation plus 1 µm radius from the nuclear edge to account for cytoplasm. For detection of RNAscope dots the FISH module v3.2.3 was used with the parameter detailed in **Supplementary Table S2**. Cells with one or more dots per probe were classified as positive for that probe.

### Patch clamp recordings

Mouse spleen NK cells were used for patch clamping within 8 hours after isolation. NK cells were seeded on a laminin-coated coverslip and allowed to attach for 5-10 min. Whole-cell patch clamping was performed by NYU Langone Health’s Ion Laboratory (RRID: SCR_021754) using an Axopatch-200B amplifier. Data were recorded with a Digidata 1550A and Clampex 10 software. Currents were low-pass filtered at 2 kHz with a 8 pole Bessel response, and data were acquired at 10 kHz (Digidata 1550A and Clampex 10.7; Molecular Devices). The bath solution consisted of (in mM) 140 NaCl, 5 KCl, 1 MgCl_2_, 1 CaCl_2_, 10 glucose, 10 HEPES, pH 7.4 adjusted with NaOH. Patch pipettes were made using borosilicate capillaries (1.5 mm O.D.; World Precision Instruments, Sarasota, Florida) and had resistances of ~4 MΩ when filled with a solution consisting of (in mmol/L) 130 KCl, 5 EGTA, 1 CaCl_2_, 1 MgCl_2_, 10 HEPES, 0.1 Mg-ATP, 1 Na_2_-UDP, pH 7.2 adjusted with KOH. From a holding potential of −70 mV, 200 ms voltage steps were applied between -140 to 50 mV (10 mV increments) at 2 s intervals. Recordings were not corrected for the liquid junction potential (estimated to be 9.4 mV). Currents were calculated as the current density (pA/pF) by dividing the current by the cell capacitance. Data were analyzed using pClamp software and custom scripts written in Python 3.0.

### Measurement of intracellular Ca^2+^

Freshly isolated mouse splenic NK cells were plated at a density of 3.0-4.0 × 10^5^ cells/well in black, flat-bottom, 96-well plates and loaded with 1 μM Fluo4/AM (Life Technologies) for 30 min in complete RPMI medium. NK cells were pre-incubated for 15 min in Ca^2+^-free Dulbecco’s Phosphate Buffered Saline solution (DPBS. 1 mM MgCl_2_, 10 mM HEPES, pH 7.4) with pinacidil (100 µM), TRAM-34 (10 µM), PNU-37883A (10 µM) or with solvent only (<0.1% DMSO). Fluorescence was recorded at excitation/emission wavelengths of 485 nm/525 nm using a microplate reader equipped with a dispenser unit for adding compounds during the recording (FlexStation 3, Molecular Devices). Fluorescence intensities were recorded every 2 s for the duration of the experiment. Store depletion was induced by stimulating the cells with 1 μM thapsigargin in Ca^2+^-free DPBS solution, and Ca^2+^ influx was induced by adding an equal volume of 0.5 mM Ca^2+^ DPBS solution to the cells (for a final Ca^2+^ concentration of 200 µM). The fluorescence intensity of each well was normalized (F/F0) by dividing by the average baseline fluorescence intensity value prior to addition of thapsigargin (F0).

### Drugs used

We used pinacidil (Sigma Aldrich, St. Louis, MO), TRAM-34 Sigma Aldrich), PNU-37883A (Tocris Bioscience, Minneapolis, MN), glibenclamide (Sigma Aldrich), thapsigargin (Invitrogen), PMA/Ionomycin (Biolegend), and Golgi Stop (Monensin) (BD Biosciences).

### Antibodies used

Antibodies used are shown in **Supplementary Table S1**.

### Statistical analysis

When comparing two groups we used the Student’s t-test. For multiple group comparison we used 1-way ANOVA. If overall significance was achieved, *ad-hoc* pairwise comparison was performed using the Tukey t-test, or the Dunnett’s t-test when comparisons were made to a single control. Statistical significance was assumed at a p-value <0.05.

## Results

### *Kcnj8* is expressed in subsets of NK cells

**Figure 1:**
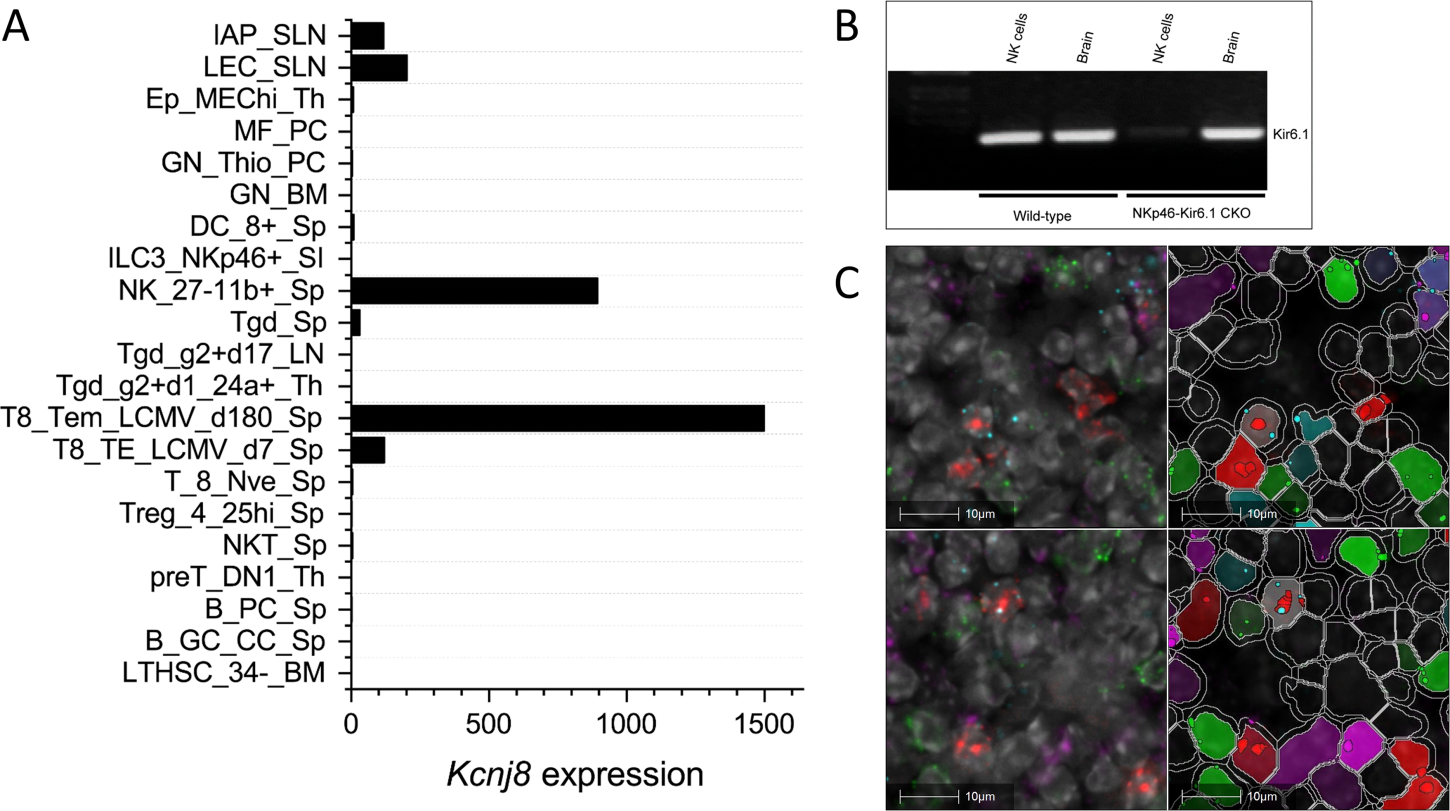
*Kcnj8* mRNA expression in NK cells. **(A)** Kcnj8 is expressed in cytotoxic effector cells in mouse immune cells. Shown are ImmGen ULI RNA seq expression data of bone marrow hematopoietic stem cells (LTHSC_34-BM), splenic germinal center centrocytes (B_GC_CC_Sp), splenic plasma cells (B_PC_Sp), thymic preT DN1 cells (preT_DN1_Th), splenic natural killer T cells (NKT_Sp), splenic CD25hi TRegs (Treg_4_25hi), splenic naïve CD8^+^ T cells (Sp T_8_Nve_Sp), splenic CD8^+^ T cells 7 days after LCMV infection (T8_TE_LCMV_d7_Sp), splenic CD8^+^ T cells 180 days after LCMV infection (T8_Tem_LCMV_d180_Sp), immature thymocytes (Tgd_g2+d1_24a+_Th), Tgd_g2+d17_LN, total splenic dgT cells (Tgd_Sp), splenic CD27^-^/CD11b^+^ NK cells (NK_27-11b+_Sp), lamina propria NKp46+ ILC3 cells (ILC3_NKp46+_SI), splenic CD8^+^ cells (DC_8+_Sp), bone marrow neutorophils (GN_BM), thio-induced peritoneal neutrophils (GN_Thio_PC), peritoneal macrophages (MF_PC), thymic medullary epithelial cells (Ep_MEChi_Th), subcutaneous lymphatic endothelial cells (LEC_SLN), and subcutaneous lymphatic pericytes (IAP_SLN). Expression values are normalized by DESeq2. Not shown are immune cell types with expression levels below 0.1. Data are from http://rstats.immgen.org/Skyline/skyline.html. **(B)** qRT-PCR data showing *Kcnj8* expression in isolated splenic NK cells and brain tissue from wild-type C57BL/6 mice and NK-cell specific NKp46-Kir6.1conditional knock-out mice. **(C)** RNAscope data of a mouse spleen. RNAscope probes used were designed to localize NK cells (*Ncr1*; red), T cells (*Cd3*; green), macrophages (*Abgre1*; magenta), and cells expressing *Kcnj8*; light blue*)*. Shown in the left are unprocessed images, with DAPI staining in gray scale. Cell segmentation was performed with DAPI as the cell marker and is shown on the right. Detected cells are outlined in white. Cells are pseudo colored based in expression of RNAscope dots. The top and bottom rows are two representative images.

Datasets from the Immunological Genome Project (ImmGen) mouse database show that *Kcnj8* is highly expressed in spleen CD27^-^/CD11b^+^ NK cell subsets and, upon viral infection, is strongly upregulated in cytotoxic CD8^+^ T cells (**Figure 1A**). We confirmed the expression of *Kcnj8* mRNA by RT-PCR in both isolated mouse NK cells and in mouse brain (**Figure 1B**). Using RNAscope analysis, we found *Kcnj8* expression in 4.4% of cells in the mouse spleen (**Figure 1C** and **Supplementary Figure S2**). Only a subset of *Ncr1*-postive NK cells (11.1%) had *Kcnj8* RNAscope dots. The quantitative RNA Scope data are in **Supplementary Table S3**. These data show that around 4% of all splenocytes express *Kcnj8*. *Kcnj8* dots are present in around 9-11% each of NK cells, CD3^+^ T cells and macrophages. This manuscript focuses on NK cells. These data show that *Kcnj8* is not expressed in all of the splenic NK cells in naïve mice.

### *Kcnj8* is expressed in mature and educated NK cells

**Figure 2:**
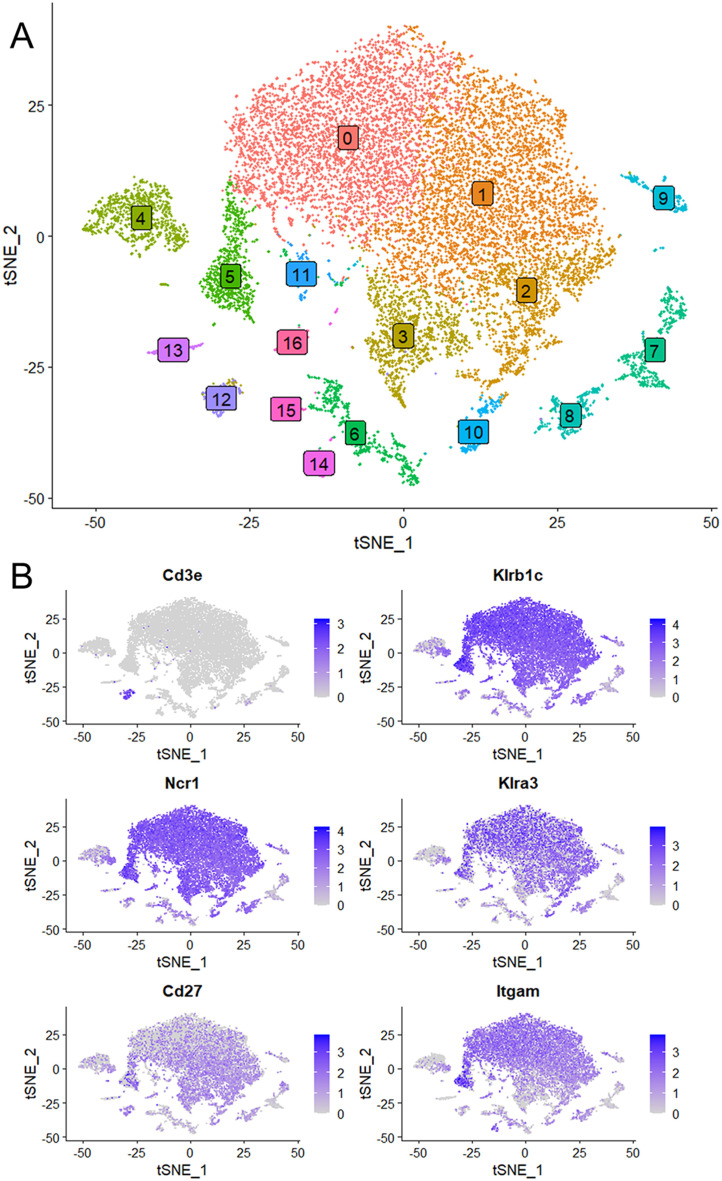
Single cell RNA seq data were obtained using NK cells of mouse spleen that were isolated using a negative selection method. Data analysis was performed with the Seurat package. **(A)** Sixteen separate cell clusters were identified by graph-based clustering algorithm. Non-linear dimensional reduction was performed and data are displayed as t-distributed Stochastic Neighbor Embedding (tSNE) plots. **(B)** Marker feature tSNE plots show lack of expression of *CD3e* (CD3E) in populations 0 to 3, and expression of the NK cell markers *Klrb1c* (NK1.1), *Ncr1* (NKp46), *Cd27* (CD27) and *Itgam* (CD11b). Also shown is high expression of *Klra3* (Ly49C) in mature NK cells (population 0).

To investigate *Kcnj8* expression in specific NK cell populations, we used a single cell RNA seq (scRNA seq) approach. NK cells were isolated from mouse spleens using a negative selection method, which largely excludes other cell types. Pooled cells from 3 mice were subjected to scRNA seq and analyzed using the Seurat package. A total of 15,878 cells were analyzed which yielded 16 individual cell clusters (**Figure 2A**). Clusters 0, 1, 2, 3 and 5 (77.5% of the total splenocytes) were NK cells, based on the expression of NK marker genes *Klrb1c* (NK1.1), *Ncr1* (NKp46), *Cd27* (CD27) and *Itgam* (CD11b), and the lack of the T-cell marker gene *Cd3ϵ* (**Figure 2B**). The remaining ~25% of cells included minor populations of B cells, macrophages, NKT cells, CD8^+^ T cells, and other immune cells (**Supplementary Table S4**).

**Figure 3:**
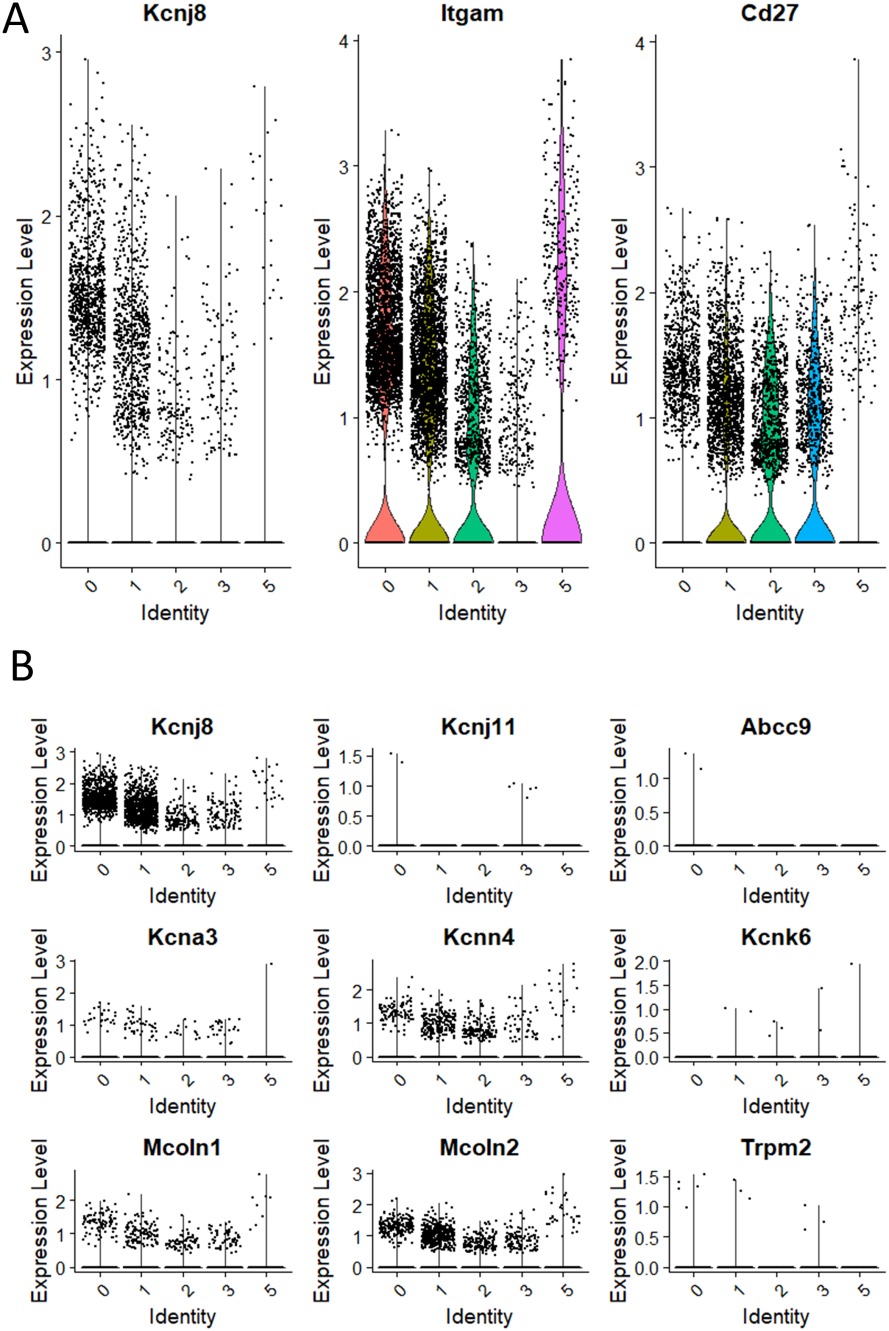
*Kcnj8* mRNA expression is highest in the mature CD11b^+^ NK cells in the mouse spleen. These data focus on populations 0 to 3 that are enriched in NK cell makers, identified in **Figure 2**. **(A)** Single cell violin plots are shown for *Kcnj8* (Kir6.1), and the NK cell maturity markers *Itgam* (CD11b), and *Cd27* (CD27). **(B)** Single cell violin plots of the scRNA seq data were generated to show the expression in populations 0 to 3 of ion channel genes *Kcnj8* (Kir6.1), *Kcnj11* (Kir6.2), *Abcc9* (SUR2), *Kcna3* (Kv1.3), *Kcnn4* (Kv1.3), *Kcnk6* (TWIK-2), *Mcoln1* (TRPML1), *Mcoln2* (TRPML2), and *Trpm2* (TRPM2). Note that *Abcc8* (SUR1) had no gene counts in this dataset.

We next focused on the major cell populations 0, 1, 2, 3 and 5 that express the NK cell markers. As maturity progresses, NK cells transition through CD27^+^/CD11b^-^, CD27^+^/CD11b^+^ and CD27^-^/CD11b^+^ subsets, becoming increasingly more cytotoxic during this progression (3). By the expression of these maturity markers, we identified populations 0 to 3 respectively to represent CD27^low^/CD11b^high^, CD27^high^/CD11b^high^, CD27^high^/CD11b^low^ and a minor population of CD27^low^/CD11b^low^ (**Figure 3A**). Cluster 5 is similar to cluster 2 and is quite advanced in the NK-cell developmental trajectory because they express *Itgam* (CD11b) and *Klra3* (Ly49C). Thus, population 3 is the least mature, followed by populations 2, 1 and 0. Interestingly, *Kcnj8* tracked the expression of CD11b, demonstrating that its expression is highest in the mature NK cells (**Figure 3B**).

We compared the expression of *Kcnj8* to other ion channels that have previously been identified in NK cells (**Figure 3B**). The expression of *Kcnj8* (Kir6.1) far exceeds those of K^+^ channels, including *Kcnj11* (Kir6.2), *Abcc9* (SUR2), *Kcna3* (Kv1.3), *Kcnn4* (Kv1.3), *Kcnk6* (TWIK-2), *Mcoln1* (TRPML1), *Mcoln2* (TRPML2), and *Trpm2* (TRPM2). *Abcc8* (SUR1) was not present in this NK cell dataset. High expression of *Kcnj8* in CD27^low^CD11b^high^ splenic NK cells can also be observed by reexamining data in public databases (**Supplementary Figure S3**) and other independent reports observed high expression of *Kcnj8* in mature mouse NK cell subsets (32–34), including educated NK cells (36, 37).

**Figure 4:**
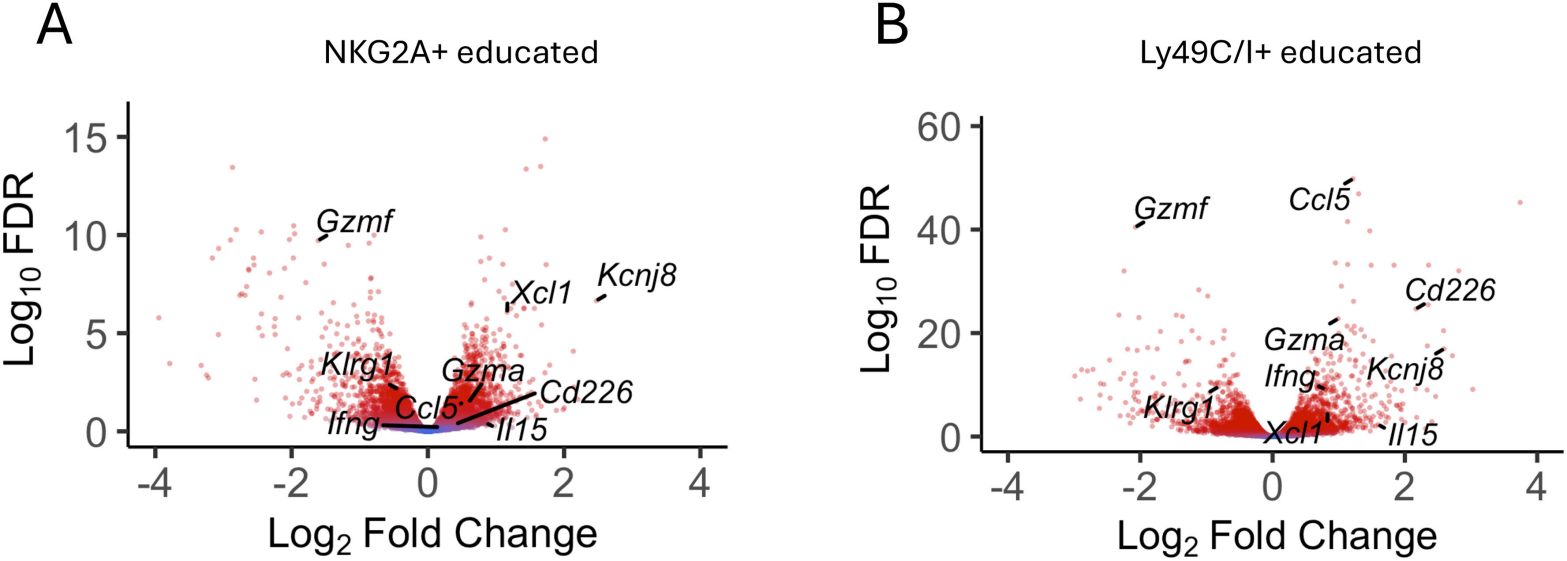
*Kcnj8* is expressed at higher levels in mature, educated uterine NK cells. Volcano plot showing selected DEGs in NKG2A+ [**(A)**, n=9] and LY49C/I+ [**(B)**, n=6] educated uNK compared to uneducated uNK cells (LY49C/I- NKG2A- n=9). Red color indicates statistical significance (FDR<0.05). Fold change expressed in log2 relative to uneducated uNK cells. A list of all DEG with their FC and FDR is accessible in **Supplementary Tables S5A** and **S5B**.

Uterine NK cells follow a different developmental trajectory from that of peripheral blood NK (pbNK) cells in both humans and mice. To analyze transcriptional changes downstream of educated uNK cells, we purified NKG2A^+^ and Ly49C/I^+^ uNK cells and analyzed differential gene expression (DEG) by comparing their transcriptomes with that of purified, uneducated NKG2A^-^Ly49C/I^-^ uNK cells. The data show that *Kcnj8* is expressed at elevated levels in NKG2A^+^ or Ly49C/I^+^ educated uNK cells, alongside known markers of NK-cell education such as *Cd266* (DNAM-1). Other highly expressed DEG include cytokines and chemokines (e.g: *Ccl5*, *Il15*), effector molecules (e.g. *Ifng*, *Xcl1, Gzmf*), transcription factors, and signaling molecules (**Figure 4**). Overall, consistent with the data obtained with splenic NK cells, educated uNK cells expressed elevated levels of *Kcnj8*.

### K_ATP_ channel recordings in mouse splenic NK cells

**Figure 5:**
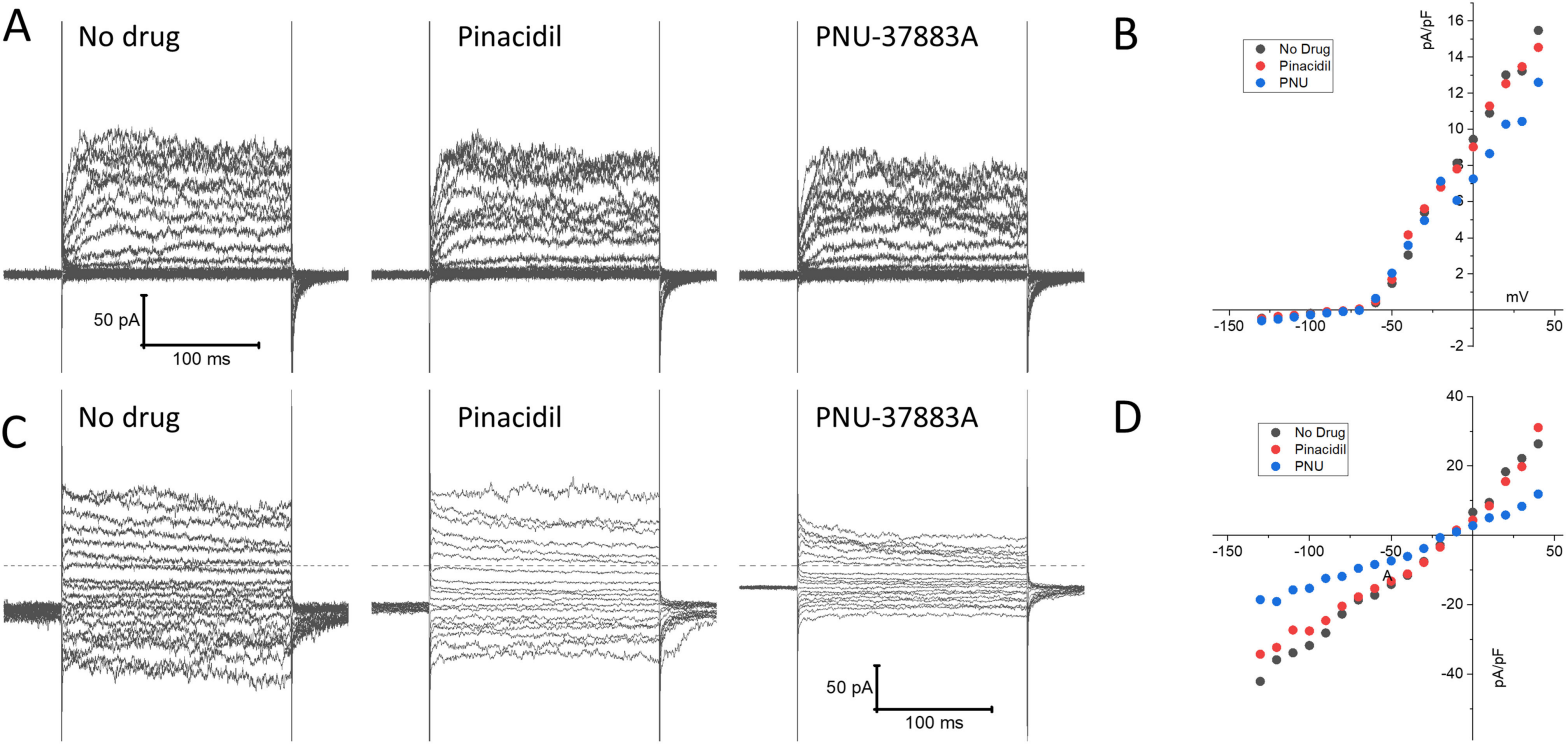
Patch clamp current recordings in isolated mouse spleen NK cells. NK cells, isolated from wild-type mouse splenocytes by negative selection, were subjected to whole-cell patch clamp recordings. **(A)** An example of recordings of a cell that exhibited time-dependent currents upon depolarization with little inward rectification. Shown on the left are recordings made in the absence of drug, 3 min after application of 100 µM pinacidil, and 3 min after application of 10 µM PNU-37883A (in the continued presence of pinacidil). **(B)** Depicted is the current, measured at the end of the voltage step, as a function of applied voltage of this cell, demonstrating pronounced outward rectification. **(C)** A typical example of a cell with a distinctly different current profile, that showed little time dependence and the presence of strong inward currents upon hyperpolarization. **(D)** The current-voltage relationship of this cell was essentially linear, and the current was strongly blocked by PNU-37883A.

We next performed patch clamp experiments to determine whether K_ATP_ channel currents can be measured in NK cells. NK cells were isolated from mouse spleen using a negative selection method and subjected to whole-cell patch clamping on the same day. Typical whole-cell patch clamp recordings are shown in **Figure 5**. Two types of current profiles were present in these cells. In 6 of 10 cells, there was a time-dependent activation of currents during depolarization and the currents were strongly outward rectifying (**Figures 5A, B**). The time-dependent profile is indicative of the presence of a voltage-gated K^+^ current. We used PNU-37883A in these experiments since it is a Kir6.1 pore blocker (35, 36). Neither the K_ATP_ channel opener pinacidil (100 µM) nor PNU-37883A (10 µM) had significant effects on the recorded currents. Four of the 10 cells, by contrast, exhibited little time dependence and the currents were relatively linear as a function of voltage (**Figures 5C, D**). Pinacidil had no appreciable effects on these currents, but PNU-37883A blocked both inward and outward currents. At -100 mV, for example, PNU-37883A decreased the current by -46 ± 6.7% (n=4; p<0.01; paired-t-test). These data are consistent with the presence of a Kir6.1-containing K_ATP_ channel in a subset of NK cells that is sensitive to the channel blocker PNU-37883A.

### *Kcnj8* does not regulate degranulation or INF-γ release in NK cells

**Figure 6:**
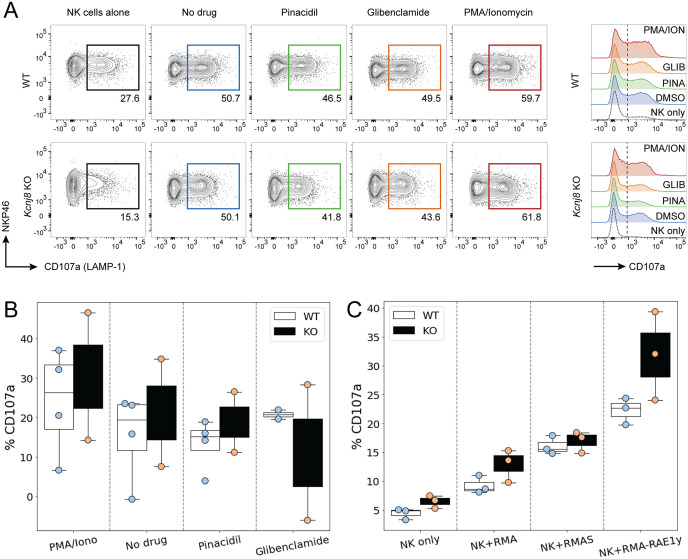
NK cell degranulate effectively in both WT and NK-cell specific *Kcnj8* KO mice. **(A)** Flow cytometry gating strategy to detect CD107a+ (degranulating) NK cells in a co-culture of NK cells and YAC-1 cells. NK cells, isolated from mouse spleen, were co-cultured for 2 h with YAC−1 target cells in the presence of the K_ATP_ channel opener (30 µM pinacidil), a K_ATP_ channel blocker (1 µM glibenclamide). Phorbol 12-myristate 13-acetate (PMA; 80 nM) and ionomycin (1.3 µM) was used as a positive control. The no drug group contained solvent only (<0.01% DMSO). **(B)** Summary data of all experiments using the K_ATP_ channel drugs. **(C)** Spleen NK cells isolated from WT or NK-specific *Kcnj8* KO mice were co-cultured with or without the target cell lines, RMA, RMA-S and RMA-RAE1y. Flow cytometry was performed to detect the percentage of CD107a+ NK cells (as a surrogate marker of degranulation). The data are from 3 separate experiments.

We first assessed the effects of pharmacological K_ATP_ channels intervention on NK cell degranulation. Surface expression of CD107a (LAMP-1) was measured by flow cytometry as an index of degranulation. WT mice were compared with tamoxifen-inducible NK cell-specific *Kcnj8* deficiency. Mice were pretreated with poly I:C, a synthetic dsRNA that strongly stimulates innate immunity. NK cells were isolated from the spleens and were co-incubated for 2 h with YAC−1 target cells in the presence of the K_ATP_ channel opener (30 µM pinacidil), a K_ATP_ channel blocker (1 µM glibenclamide). As a positive control, cells were incubated with a combination of phorbol 12-myristate 13-acetate (PMA; 80 nM) and ionomycin (1.3 µM), which is known to stimulate NK cell degranulation (37). A negative control consisted of the drug solvent only (<0.01% DMSO). Neither the K_ATP_ channel opener nor the blocker affected degranulation in this *in vitro* assay (**Figures 6A, B**). Moreover, there was no significant difference when comparing the WT and KO groups (2W ANOVA; p = 0.794).

We next assessed the impact of *Kcnj8* on NK cell degranulation against different types of target cells. We used three types of lymphoma cells: parental RMA cells expressing MHC class I molecules; mutant RMA-S cells expressing very low MHC class I molecules and mutant RMA-Rae1γ cells expressing MHC class I molecules along with the NKG2D ligand. The RMA−S cells and RMA-Rae1γ cells elicit stronger NK cell activation than the RMA cells (38, 39). We compared wild-type mice with mice constitutively deficient of *Kcnj8* in NK cells. In both groups, NK cells were pre-stimulated by injecting poly(I:C). After 2 days, NK-enriched splenocytes were co-incubated with either RMA, RMA-S or RMA-Rae1γ cells. The percentage of CD107a^+^ degranulating NK cells in response to each of the three RMA cell type was quantified after 2 hours by flow cytometry (n=3-4, repeated twice). As expected, NK cells responded with higher CD107a^+^ cell percentages to the RMA-S and RMA-Rae1γ cells when compared to RMA cells (**Figure 6C**). There was, however, no statistical difference when comparing the genotypes (2W ANOVA; p=0.860). When gating on the different CD27^+^/CD11b^+^ subsets, there were also no significant genotype differences in the percentage of CD107a^+^ cells within each subset (data not shown).

We also examined INF-γ release from splenic NK cells incubated with IL-15, IL-15 plus IL-18, or with the positive control PMA/ionomycin. As expected, IL-18 and PMA/ionomycin led to significant elevation of INF-γ in the cell culture media (assessed by ELISA) (p<0.01; 2W ANOVA). However, there were no significance differences by genotype (p=0.32; 2W ANOVA; **Supplementary Figure S4**), demonstrating that INF-γ release was unaffected by *Kcnj8* under these experimental conditions.

Taken together, our data show that NK cells can degranulate effectively in the absence of *Kcnj8*. Moreover, INF-γ release stimulated by IL-18 was unaffected by the absence of *Kcnj8*. It remains to be investigated whether these processes would be affected during metabolic stress (when K_ATP_ channels are expected to open), such as the hypoxic environment of a solid tumor.

### *Kcnj8* is not required for tumor rejection by NK cells

**Figure 7:**
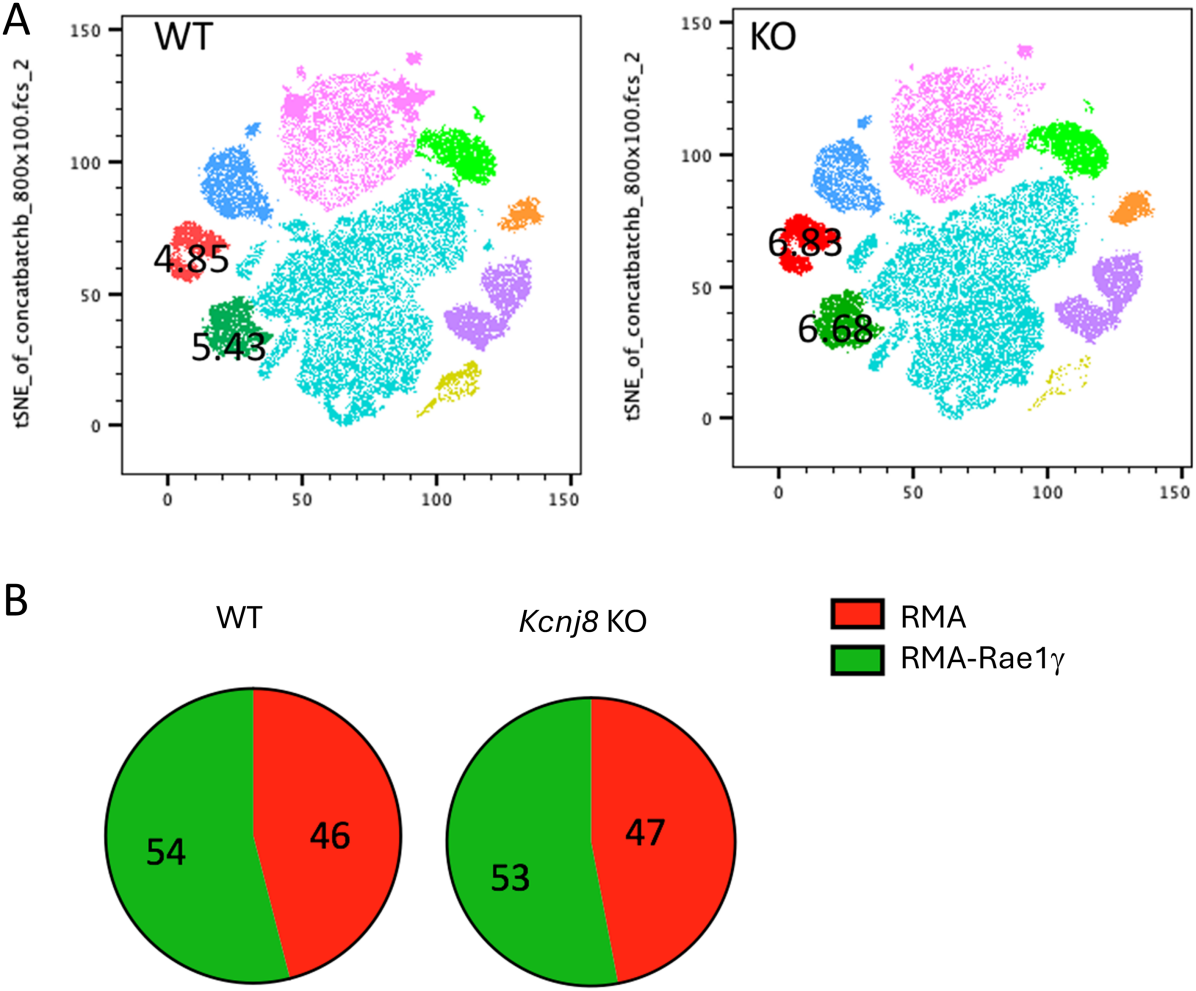
NK cells reject tumor cells equally *in vivo* in WT and NK- specified *Kcnj8* KO mice. Mice were injected I.P. with a mixture of RMA-S, RMA and RMA-RAE1y cells, pre-stained with fluorescent markers for later visualization and identification. The RMA-Rae1γ cells are preferential NK cell targets due to high expression of MHC class I molecules. After 48 h, cells were isolated from the peritoneum and subjected to flow cytometry to quantify the relative amounts of target cells. **(A)** Cells from the peritoneal fluid of four WT and four NK- specified *Kcnj8* KO mice were downsampled to equal cell counts of viable cells per group (40.000 cells per group) in FlowJo and concatenated to a single file prior to unbiased dimensionality reduction approach, visualized as tSNE plots. The clusters were determined by protein expression levels of forward and side scatter (FSC & SSC), H2Kb, CD3, CFSE, FarRed, NK1.1 and CD69. RMA-S cells were not detected. The RMA (red) and RMA-RAE1y (green) cell populations each comprised about 5% of the number of cells in both the WT and KO mice. **(B)** Depicted is the percentage of RMA and RMA-RAE1y cells found in peritoneal fluid of WT and KO mice. Experiment was repeated twice with similar results.

In a next experiment, we tested the efficiency of NK cells to reject tumor cells *in vivo*. We administered an equal mix of RMA, RMA-S and RMA-Rae1γ cells intraperitonially to control mice and mice constitutively deficient of *Kcnj8* in NK cells. After 48 hours, tumor cells were harvested and quantified. Very few MHC-deficient RMA-S cells were present in both groups, suggesting they had been rejected by NK cells upon missing-self recognition (n=3-4, repeated twice). We focused therefore on the ratio of RMA to RMA-Rae1γ cells. By flow cytometry, we detected several different cell populations in the peritoneal cavity (**Figure 7A**). The RMA and RMA-Rae1γ cells tumor cells were recovered in approximately the same ratio in the control and KO mice, suggesting no obvious defect in recognition of induced self in the KO mice (**Figure 7B**). These results suggest that the *in vivo* anti-tumor NK-cell response is effective in KO mice. Therefore, *Kcnj8* deficiency does not seem to affect overall NK-cell cytotoxicity under these specific conditions or tumor cells tested.

### No role for K_ATP_ channels in store-operated Ca^2+^ entry in mouse NK cells

**Figure 8:**
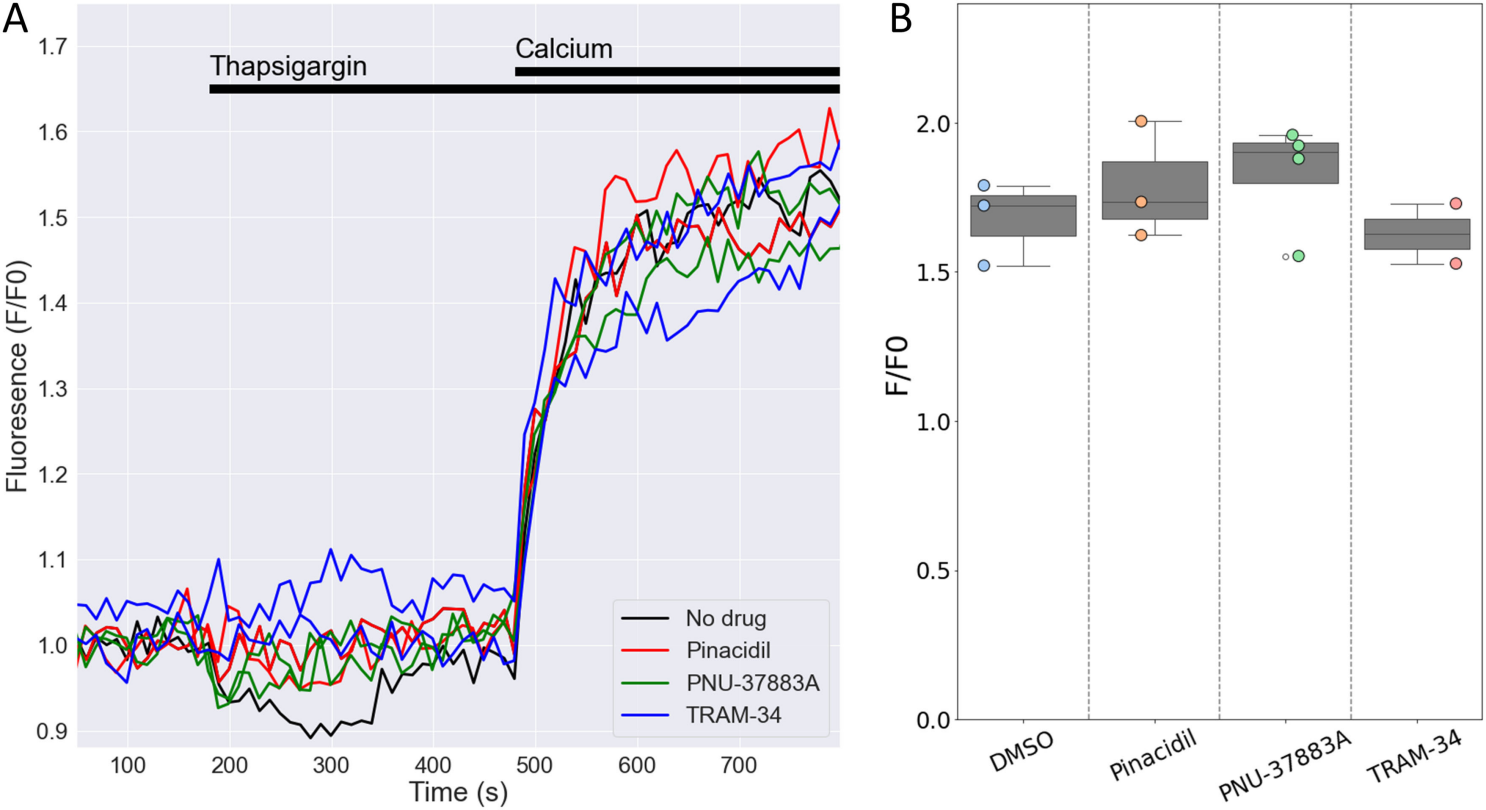
Openers or blockers of K_ATP_ channels do not affect store-operated Ca^2+^ entry (SOCE) in mouse NK cells. Cytosolic Ca^2+^ of isolated splenic NK cells was recorded with Fluo4/AM. **(A)** Depicted are representative F/F0 traces in different wells of the same experiment with NK cells pretreated for 15 min with pinacidil (100 µM), TRAM-34 (10 µM), PNU-37883A (10 µM) or with no drug (<0.1% DMSO). Store depletion was accomplished by 1 μM thapsigargin and SOCE was initiated by adding 200 µM CaCl_2_ to the external solution. **(B)** Summary data of experiments performed on different days (n=3 mice). p=0.84 with 1W-ANOVA.

Degranulation is dependent on store-operated Ca^2+^ entry (SOCE) into human NK cells (20), The Ca^2+^-activated K^+^ channel KCa3.1 regulates SOCE by preventing SOCE-induced membrane depolarization, thereby maintaining Ca^2+^ influx (15). Given the high expression of the *Kcnj8* in mouse cytotoxic NK cells, we tested whether K_ATP_ channels may have a similar role in SOCE in mouse NK cells. We used a well-described protocol to deplete Ca^2+^ from the endoplasmic reticulum (ER) with the sarco-endoplasmic reticulum Ca^2+^ ATPase (SERCA) inhibitor thapsigargin (TG), which triggers SOCE through Ca^2+^ release-activated Ca^2+^ (CRAC) channels (40). We recorded SOCE by measuring cytosolic Ca^2+^ with Fluo-4/AM while depleting the ER stores, and following the addition of 200 µM extracellular Ca^2+^ to initiate SOCE (**Figure 8A**). The magnitude of the SOCE response was unaffected by the K_ATP_ channel opener pinacidil or the Kir6.1-blocker PNU-37883A (**Figures 8A, B**). Blocking KCa3.1 with TRAM-34 also had no effect on SOCE, which is consistent with the low *Kcnn4* (KCa3.1) mRNA counts in the mouse spleen NK cells (**Figure 3B** and **Supplementary Figure S3**). These data suggest that neither KCa3.1 nor K_ATP_ channels contribute to SOCE in mouse splenic NK cells.

### Effects of *Kcnj8* deficiency on the transcriptome of NK cells

**Figure 9:**
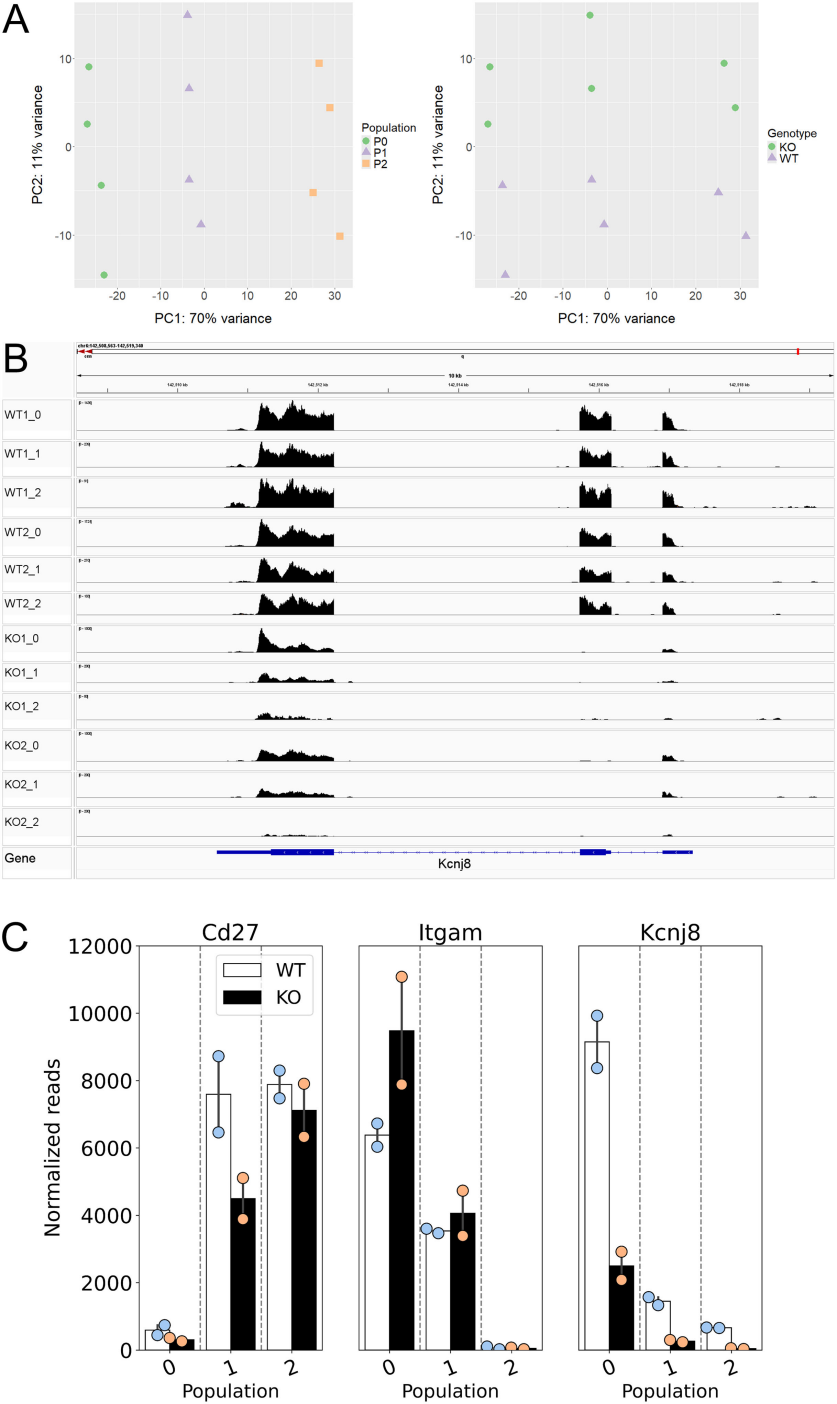
Data of a bulk RNA seq experiment performed with mouse splenic cells, NK cells isolated by cell sorting by first using NK markers NK1.1 and NKp46, then NK cell maturity markers CD27 and CD11b (n=2 each of WT and tamoxifen-induced NK cell-specific *Kcnj8* KO mice). Normalized reads and differentially expressed genes were calculated using the DEseq2 package. Normalized gene expression was averaged within populations. **(A)** Principal component analysis demonstrates segregation of the three populations 2, 1 and 0 (respectively CD27^+^/CD11b^-^, CD27^+^/CD11b^+^ and CD27^-^/CD11b^+^). **(B)** Mapping of RNA seq reads against the mouse mm10 genome was performed with IGV (Integrative Genomics Viewer). Note that the *Kcnj8* gene at the bottom of the panel is oriented in the reverse direction (3’ to 5’). **(C)** Normalized *Cd27* (CD27), *Itgam* (CD11b), and *Kcnj8* (Kir6.1) reads in the three different populations for wild-type (WT) and mice with NK cell-specific *Kcnj8* deficiency (p<0.05 with the Mann-Whitney Rank Sum Test). Note that, although *Kcnj8* reads remain in the KO mice, they are from exons 1 and 3 as shown in panel **(B)**.

In order to better understand the consequences of NK cell *Kcnj8* deficiency, we performed a bulk RNA seq experiment of freshly isolated splenocytes, with NK cells isolated by cell sorting using CD27 and CD11b antibodies. RNA seq experiments were performed with sorted NK cells from wild-type mice and tamoxifen-inducible NK cell-specific *Kcnj8* deficiency (n=2 each). The expectation was that these data may point to underlying transcriptional changes and pathways affected by the absence of *Kcnj8*. The read counts were variable in the immature CD27^-^/CD11b^-^ population due to low cell yields and these data were therefore excluded from analysis. A principal component analysis of the remaining three populations (**Figure 9A**) demonstrated that NK cell populations CD27^+^/CD11b^-^, CD27^+^/CD11b^+^ and CD27^-^/CD11b^+^ (respectively populations 2, 1 and 0 in order of maturity) segregated separately, indicative of transcriptional differences between these cell types. The variance between genotypes were small, demonstrating that *Kcnj8* deficiency led to specific transcriptional changes. When mapping the individual reads to *Kcnj8* on the mouse genome, it is clear that reads within exon 2 are largely absent in the *Kcnj8* knockout mice, as expected from the knockout strategy (**Figure 9B**). The normalized reads for *Cd27* (CD27) and *Itgam* (CD11b), which verifies the nature of the NK cell populations are shown in **Figure 9C**. Also shown are the normalized reads of *Kcnj8* in the different groups, which makes it evident that the mature CD27^-^/CD11b^+^ NK cells express significantly elevated levels of *Kcnj8* compared to the less mature CD27^+^/CD11b^+^ and CD27^+^/CD11b^-^ populations in WT mice. Given that *Kcnj8* expression is so much elevated in the CD27^-^/CD11b^+^ population, we performed a differential gene expression analysis in this population by comparing the WT and KO genotypes. A total of 29 genes were differentially expressed, with 16 upregulated and 13 downregulated (**Table 1**). A gene ontology analysis reveals affected biological processes to be involved that include phospholipase C activity, cytokine production and JAK-STAT signaling (**Supplementary Figure S5**).

**Table 1:** Differentially expressed genes in the CD27^-^/CD11b^+^ population of NK cells.

Gene	Name	baseMean	log2FoldChange	lfcSE	pvalue	padj
*Uty*	ubiquitously transcribed tetratricopeptide repeat containing, Y-linked	224.1	-9.54	1.38	9.47E-12	1.59E-08
*Kdm5d*	lysine demethylase 5D	296.1	-9.12	1.04	1.87E-17	4.05E-14
*Capn11*	calpain 11	33.5	-8.60	2.94	3.37E-07	0.000243
*Ddx3y*	DEAD box helicase 3, Y-linked	1034.6	-8.14	0.53	6.78E-54	1.03E-49
*Ighg2c*	immunoglobulin heavy constant gamma 2C	747.5	-6.69	0.51	4.14E-40	2.09E-36
*Slpi*	secretory leukocyte peptidase inhibitor	54.4	-4.31	0.98	4.92E-07	0.00031
*Kit*	KIT proto-oncogene receptor tyrosine kinase	946.6	-3.55	0.41	8.97E-20	2.26E-16
*Dtx1*	deltex 1, E3 ubiquitin ligase	328.1	-2.91	0.43	3.01E-13	5.69E-10
*H2-Ab1*	histocompatibility 2, class II antigen A, beta 1	346.1	-2.89	0.56	1.05E-08	1.06E-05
*Cd74*	CD74 antigen (invariant polypeptide of major histocompatibility complex, class II antigen-associated)	547.9	-2.86	0.48	1.22E-10	1.84E-07
*Ltb*	lymphotoxin B	198.1	-2.51	0.53	7.65E-08	6.44E-05
*Hk3*	hexokinase 3	201.0	-2.28	0.47	5.25E-08	4.96E-05
*Il7r*	interleukin 7 receptor	216.8	-2.21	0.51	4.87E-07	0.00031
*Adgrg5*	adhesion G protein-coupled receptor G5	564.4	-1.83	0.38	5.91E-08	5.26E-05
*Kcnj8*	potassium inwardly-rectifying channel, subfamily J, member 8	4795.2	-1.71	0.32	2.59E-09	3.02E-06
*Tcf7*	transcription factor 7, T cell specific	1320.5	-1.59	0.36	3.13E-07	0.000237
*Rhpn1*	rhophilin, Rho GTPase binding protein 1	2733.8	1.03	0.36	0.000125	0.045062
*Tigit*	T cell immunoreceptor with Ig and ITIM domains	707.1	1.31	0.37	1.58E-05	0.007478
*Fam234b*	family with sequence similarity 234, member B	434.6	1.55	0.38	1.60E-06	0.000837
*Serpinb9b*	serine (or cysteine) peptidase inhibitor, clade B, member 9b	10862.6	1.58	0.29	2.57E-09	3.02E-06
*Frmd5*	FERM domain containing 5	56.8	2.48	0.80	6.94E-05	0.028408
*Gp6*	glycoprotein 6 platelet	81.3	2.94	0.75	3.72E-06	0.001878
*Rab4a*	RAB4A, member RAS oncogene family	76.8	3.30	0.73	2.76E-07	0.00022
*Esr1*	estrogen receptor 1 (alpha)	829.5	3.32	0.38	5.52E-20	1.67E-16
*Emp1*	epithelial membrane protein 1	1643.9	4.80	0.44	1.78E-29	6.73E-26
*Firre*	functional intergenic repeating RNA element	62.9	4.95	0.92	6.44E-09	6.96E-06
*Sh2d1b2*	SH2 domain containing 1B2	30.0	5.75	1.76	0.000147	0.049454
*H2af-ps2*		31.6	6.02	1.61	4.89E-05	0.021788
*Xist*	inactive X specific transcripts	825.3	8.16	0.60	1.10E-42	8.34E-39

Bulk RNA seq data are from mouse splenocytes, sorted by expression of CD27 and CD11b. DEseq2 analysis was performed to compare wild-type with Kir6.1 KO mice (n=2 each). Shown here are differentially expressed genes, defined by a log2fold value larger than 1.0 or smaller than -1.0; with an adjusted p-value < 0.05.

### *Kcnj8*-deficient NK cells fail to reach full maturity

**Figure 10:**
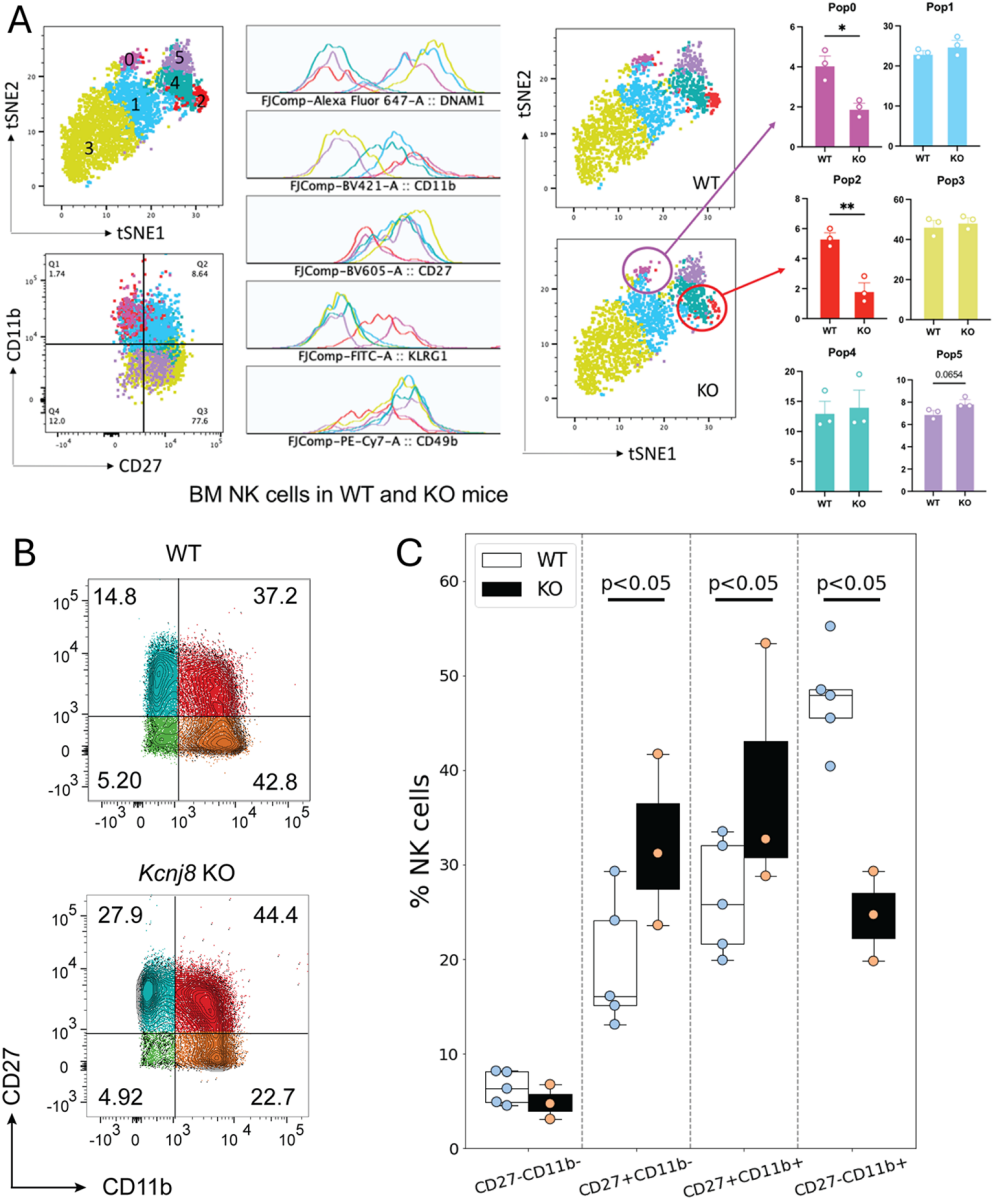
NK cell maturation in bone marrow and spleen of NK-specific *Kcnj8* KO and WT mice. **(A)** NK cells, isolated from bone marrow, were subjected to flow cytometry. Data of WT and constitutive NK-cell specific *Kcnj8* deficient mice are contrasted. A tSNE plot of differentially expressed proteins identified six populations of NK cells with differential protein expression (population 0 – population 5). NK maturation markers in each population is shown. WT is shown on the top and KO is the bottom. The frequency of each of the NK cell populations are compared between WT and KO mice. *p<0.05; **p<0.001 using a Student’s t-test. **(B)** Isolated spleen NK cells from WT mice, or mice with tamoxifen-induced NK-cell specific *Kcnj8*-deficiency, were subjected to flow cytometry. NK cells were selected based on expression of NK1.1 and NKp46. Cells were further selected based on expression of CD27 and CD11b. **(C)** Summary data from 3-4 separate mice in each group is depicted as bar graphs. p<0.05 with 2W-ANOVA, followed by a Dunnett’s t-test.

Among the downregulated genes in the CD27^-^/CD11b^+^ NK cells in the *Kcnj8* KO were *Il7r*, *Ltb* (Lymphotixin beta), and *Kit* proto-oncogene receptor tyrosine kinase (c-Kit, or CD117). These all have key roles in NK cell development (41–43). We therefore investigated the possibility that *Kcnj8* may be involved with NK cell maturity, marked by the expression of surface markers CD27 and CD11b in bone marrow and spleen (3). We compared wild-type mice with mice with NK-cell specific *Kcnj8* ablation. First, we stained bone marrow NKp46^+^/NK1.1^+^ NK cells of control and constitutive NK-specific *Kcnj8*-deficient mice (KO, n=3 each) with CD27, CD11b, DNAM-1, KLRG1 and CD49b to define discrete developmental stages in both groups. The analysis showed a significant reduction of the most mature NK cell subset CD27^-^/CD11b^+^ subset (**Figure 10A**), and a reduction in mature KLRG1^+^ NK cells (**Supplementary Figure S6**), whereas other populations were unchanged. We repeated this experiment with isolated splenic NK cells using wild-type and tamoxifen-induced NK cell-specific *Kcnj8* deficiency (**Figures 10B, C**). As expected, the number of double negative (DN; CD27^−^/CD11b^−^) immature NK cells was very low in the spleen. The major NK cell populations (in order of maturity) of wild-type mice were CD27^+^/CD11b^−^, CD27^+^/CD11b^+^ and CD27^-^/CD11b^+^ NK cells. The total number of splenic NK cells was 50,000 and 40,000 respectively in WT and KO. In the NK cell-specific *Kcnj8* KO mice, there was a ~50% decrease of the most mature CD27^-^/CD11b^+^ NK cells, with a corresponding increase in the immature CD27^+^/CD11b^-^ cell population (**Figure 10C**).

## Discussion

In this study, we found that *Kcnj8* is highly expressed in mature, educated NK cells. Within the most mature, CD27^-^/CD11b^+^ NK cell subset, genes with known roles in NK cell development are differentially expressed in the NK cell-specific *Kcnj8* KO mice, in which the developmental trajectory is significantly impaired. Overall, these data point to a previously unexplored cell-intrinsic role of this K_ATP_ channel subunit in NK cell development.

### Kir6.1 as a K_ATP_ channel subunit in mouse NK cells

Although there are no controlled clinical studies, there are indications in the literature that K_ATP_ channels may have a role in immunity. For example, glibenclamide, which is an anti-diabetic sulfonylurea (a K_ATP_ channel blocker), mitigates the secretion of proinflammatory cytokine production in polymorphonuclear neutrophils from diabetic patients (44). Benefit of glibenclamide was also observed in melioidosis (45). In mice, Kir6.1 (*Kcnj8*) deficiency causes an exaggerated susceptibility to lipopolysaccharide (LPS) (46). A random mutagenesis screen in mice identified the m*ayday* mutation that caused a profound susceptibility to infection by mouse cytomegalovirus (MCMV) with decreased peak cytokine responses. The *mayday* mice also had a ~20,000-fold sensitization to LPS and were hypersensitive to the lethal effects of poly(I.C) and CpG DNA (47). The *mayday* mutation was mapped to a deletion of *Kcnj8*, essentially leading to a knockout of *Kcnj8*.

We were able to record ion channels in NK cells with pharmacological profile that matches that of K_ATP_ channels. The currents were insensitive to the K_ATP_ channel opener pinacidil. The lack of effect of pinacidil on the currents can be explained if the K_ATP_ channels were constitutively open in these cells under our experimental conditions. Moreover, the Kir6.1 blocker PNU-37883A inhibited ionic current magnitude, but only in cells where the current amplitude was large. The interpretation of the patch clamp experiments is complicated by the finding that only a subset of NK cells expresses *Kcnj8*.

Functional K_ATP_ channels are formed by co-assembly of Kir6.1 (or Kir6.2) with one of the sulphonylurea receptors, SUR1 or SUR2 (21). Although mouse NK cells robustly express *Kcnj8*, the expression of *Abcc8* (SUR1) is absent and *Abcc9* (SUR2) mRNA expression is very low. Even though we can measure PNU-37883A sensitive currents in mouse NK cells with patch clamp approaches, we are open to the possibilities that PNU-37883A may not be completely specific against Kir6.1, and that Kir6.1 may have non-channel roles to regulate NK cell maturation and education. There is ample precedent for ion channel subunits that have functions beyond their role as ion channels. For example for Kv2 channels to participate in the formation of endoplasmic reticulum/plasma membrane junctions (48), the BKCa channel subunit not only forms Ca^2+^-activated K^+^ channels but also has roles in cell signaling and gene expression regulation (49), and Navβ subunits of voltage-gated Na^+^ channels that are involved in cell adhesion, particularly during development, and can influence cell migration and neurite outgrowth (50). Experiments to determine possible non-channel roles for Kir6.1 in NK cells are ongoing.

### Species differences in NK cell function and channel expression

NK cells from different species express different proteins and markers to perform the same functions (5). Differences in expression and function of ion channels and transporters between mice and humans remain to be elucidated. The negative membrane potential of most mammalian cells is determined by K^+^ channels. In NK cells, a role of the resting potential is illustrated by the finding that depolarizing NK cells with a high K^+^ concentrations results in partial inhibition of lysis (51). Moreover, a negative membrane potential is needed to maintain Ca^2+^ oscillations in NK cells following cross-linking with anti-CD16 (52). Early studies have revealed that K^+^ channels are indeed involved in the adhesion of NK cells to target cells and are crucial for lysis of target cells by NK cells from human peripheral blood (10, 11). In these studies, K^+^ currents were recorded from the NK cells. These channels were blocked by 4-aminopyridine, quinidine and by the traditional Ca^2+^ channel blockers verapamil and Cd^2+^. Moreover, the K^+^ channel blockers quinidine, Cd^2+^ and 4-AP, but not TEA, inhibited NK cell mediated killing of target cells (9, 10, 51).

At the molecular level, the expression and functional relevance of K^+^ channels is best characterized in human T lymphocytes, which express channels subunits such as KCa3.1 and Kv1.3 (8). There is evidence that these two channels may also be present in human NK cells. Indeed, expression of the voltage-gated Kv1.3 and the Ca^2+^-activated KCa3.1 K^+^ channel was confirmed in human peripheral blood NK cells (14). Interestingly, after activation of the NK cells by mitogens or tumor cells, adherent NK cells were found to preferentially upregulate KCa3.1, whereas non-adherent NK cells preferentially upregulate Kv1.3. Blockers of Kv1.3 mitigated the proliferation and degranulation of non-adherent NK cells, with minimal effects on the adherent NK cells. There is also evidence for a role of a K^+^ channel from a different class, namely the two-pore domain family. Specifically, K2p5.1 (also named TASK2) is expressed in human peripheral blood NK cells (16). An interesting observation in the latter study was K^+^ channel expression is distinctly regulated during maturation as NK cells progress from the immature CD56-/CD16- cells, to CD56^bright^/CD16-, CD56^bright^/CD16^+^ and the most cytotoxic CD56^dim^/CD16^+^ subpopulations. K2p5.1 was downregulated during maturation through these cell stages, whereas Kv1.3 was upregulated. Thus, the complement of K^+^ channels, and their roles in NK cell function, may depend on the state of the human NK cell.

Our transcriptomics analysis of the mature CD27^-^/CD11b^+^ cells indicated that a) this NK cell population expresses the highest *Kcnj8* mRNA levels by far, and b) that downregulated genes with *Kcnj8* deficiency include *Il7r* (interleukin 7 receptor or IL7R), *Ltb* (Lymphotoxin B), and *kit* (KIT proto-oncogene receptor tyrosine kinase; c-Kit, CD117). All of these genes have critical roles in NK cell development (41–43). Indeed, we found that the NK cell maturity profile is significantly impacted in the bone marrow and spleen of mice with NK cell-specific *Kcnj8* deficiency. The transcriptomics dataset can be further explored in future experiment since several other genes are either up- or downregulated by NK cell-specific *Kcnj8* deficiency in the CD27^-^/CD11b^+^ cells (**Table 1**). Repression of the Notch1 target gene *Dtx1*, for example, prevents NK-cell differentiation (53). *Tigit* (T cell immunoreceptor with Ig and ITIM domains), is upregulated with *Kcnj8* deficiency. The interaction between *Kcnj8* and *Tigit* might be an attractive candidate to explore in future experiments given the latter gene’s roles in in NK cell education, function, activation, functional heterogeneity, exhaustion, and antitumor responses (54–58).

## Data Availability

The data presented in the study are deposited in the GEO repository, accession number GSE282716.
